# Expression Analyses of Soybean VOZ Transcription Factors and the Role of *GmVOZ1G* in Drought and Salt Stress Tolerance

**DOI:** 10.3390/ijms21062177

**Published:** 2020-03-21

**Authors:** Bo Li, Jia-Cheng Zheng, Ting-Ting Wang, Dong-Hong Min, Wen-Liang Wei, Jun Chen, Yong-Bin Zhou, Ming Chen, Zhao-Shi Xu, You-Zhi Ma

**Affiliations:** 1 Institute of Crop Science, Chinese Academy of Agricultural Sciences (CAAS)/National Key Facility for Crop Gene Resources and Genetic Improvement, Key Laboratory of Biology and Genetic Improvement of Triticeae Crops, Ministry of Agriculture, Beijing 100081, China; libo708@126.com (B.L.); zhouyongbin@caas.cn (Y.-B.Z.); chenming02@caas.cn (M.C.); mayouzhi@caas.cn (Y.-Z.M.); 2 Anhui Science and Technology University, Fengyang 233100, China; zhengjiachengx2016@126.com; 3 College of Agriculture, Yangtze University; Hubei Collaborative Innovation Center for Grain Industry; Engineering Research Center of Ecology and Agricultural Use of Wetland, Ministry of Education, Jingzhou 434025, China; wangtingting11234@163.com (T.-T.W.); whwenliang@163.com (W.-L.W.); 4 College of Agronomy, Northwest A&F University/State Key Laboratory of Crop Stress Biology for Arid Areas, Yangling, Shaanxi 712100, China; mdh2493@126.com

**Keywords:** soybean, VOZ transcription factor, expression characterization, stress response, drought and salt tolerance

## Abstract

Vascular plant one-zinc-finger (VOZ) transcription factor, a plant specific one-zinc-finger-type transcriptional activator, is involved in regulating numerous biological processes such as floral induction and development, defense against pathogens, and response to multiple types of abiotic stress. Six VOZ transcription factor-encoding genes (*GmVOZs*) have been reported to exist in the soybean (*Glycine max*) genome. In spite of this, little information is currently available regarding *GmVOZs.* In this study, *GmVOZs* were cloned and characterized. *GmVOZ* genes encode proteins possessing transcriptional activation activity in yeast cells. *GmVOZ1E*, *GmVOZ2B*, and *GmVOZ2D* gene products were widely dispersed in the cytosol, while GmVOZ1G was primarily located in the nucleus. *GmVOZs* displayed a differential expression profile under dehydration, salt, and salicylic acid (SA) stress conditions. Among them, *GmVOZ1G* showed a significantly induced expression in response to all stress treatments. Overexpression of *GmVOZ1G* in soybean hairy roots resulted in a greater tolerance to drought and salt stress. In contrast, RNA interference (RNAi) soybean hairy roots suppressing *GmVOZ1G* were more sensitive to both of these stresses. Under drought treatment, soybean composite plants with an overexpression of hairy roots had higher relative water content (RWC). In response to drought and salt stress, lower malondialdehyde (MDA) accumulation and higher peroxidase (POD) and superoxide dismutase (SOD) activities were observed in soybean composite seedlings with an overexpression of hairy roots. The opposite results for each physiological parameter were obtained in RNAi lines. In conclusion, *GmVOZ1G* positively regulates drought and salt stress tolerance in soybean hairy roots. Our results will be valuable for the functional characterization of soybean VOZ transcription factors under abiotic stress.

## 1. Introduction

Plants grown in a natural environment are constantly affected by various environmental stimuli such as drought and salt stress. Upon exposure to these conditions, the expression of a wide spectrum of genes is induced, and their encoding products have been demonstrated to participate in molecular and cellular responses to diverse stresses [1,2,3,4,5,6,7,8]. Among these stress-induced genes, it should be pointed out that transcription factor genes regulate plant tolerance by activating or suppressing downstream target genes that are important for stress resistance. Thus, the transcriptional control of gene expression plays vital roles in plant responses to a range of stresses [9,10,11,12,13,14].

Vascular plant one zinc-finger proteins (VOZ) are a plant-specific transcription factor family with two members, VOZ1 and VOZ2, and exclusively exist in vascular plants and a moss, *Physcomitrella patens* [15]. VOZ transcription factors were initially identified as a kind of protein that specifically recognizes and binds to the *GCGTNx7ACGC* palindromic sequence in the regulatory region of the *Arabidopsis* V-PPase gene (*AVP1)* using yeast one-hybrid screening of a cDNA library [15]. Subsequently, the *GCGTNx7ACGC* palindromic sequence as a VOZ transcription factor binding site was reconfirmed, and several possible additional VOZ-binding *cis*-elements were also identified [16,17,18]. The examination of VOZ-binding *cis*-elements revealed that VOZ transcription factors might have pivotal roles in flowering. It has also been demonstrated that VOZ1/2 as phytochrome B-interacting transcription factors played redundant roles in regulating the transition from vegetative growth to flowering by up-regulating *FLOWERING LOCUS T* (*FT*) and down-regulating *FLOWERING LOCUS C* (*FLC*) expression in *Arabidopsis* [16,19]. On the other hand, VOZ1/2 has been shown to promoter flowering primarily by interacting with and modulating the function of CONSTANS (CO), whereas this is independent of *FLC* in *Arabidopsis* [17].

In addition to roles in flowering, it was documented that VOZ transcription factors as transcriptional regulators repress plant tolerance to abiotic stresses such as freezing, drought, and high temperature, but activate the resistance to biotic stresses including bacterial and fungal pathogens. For example, the overexpression of *VOZ2* impaired transgenic plants freezing and drought stress tolerance but increased resistance to a fungal pathogen, *Colletotrichum higginsianum*, in *Arabidopsis* [20]. In *Arabidopsis*, voz1voz2 double mutants exhibited enhanced tolerance to freezing and drought stress but increased resistance to *C. higginsianum* and a bacterial pathogen, *Pseudomonas syringae* [21,22]. Recently, it was found that VOZ1 and VOZ2 mediated high-temperature stress response by independently suppressing the expression of DREB2C or DREB2A in *Arabidopsis* [23,24]. In contrast to freezing and drought stress, VOZ transcription factors play opposite roles in response to salt stress. As reported previously, VOZ1/2 could either directly or indirectly regulate the transcript level of a great number of stress-responsive genes, thereby conferring salt tolerance in *Arabidopsis* [18]. Moreover, the role of VOZ transcription factors in tolerance to iron-deficiency stress was also investigated in *Arabidopsis*, but the findings showed that VOZ1/2 did not play a significant physiological role in the iron-deficiency response [22]. 

Since the VOZ transcription factor was first reported [15], studies on its expression and functional validation were mainly concentrated on the model plant, *Arabidopsis*, but other species have rarely been investigated. Six VOZ transcription factors were demonstrated to exist in the soybean genome [25]. According to the Plant Transcriptional Regulatory Map (PlantRegMap) database [26], the VOZ transcription factor family has more members in soybean than in many other species, which suggests that soybean VOZ transcription factors may have more complex functions and participate in more regulatory mechanisms. Given the potential importance of VOZ transcription factors in growth, development, and the response to stress in soybean, we analyzed the sub-cellular localization and transcriptional activation activity of soybean VOZ family gene-encoded proteins, the expression profiles of *GmVOZs* in different soybean tissues and the response to dehydration, salt, and salicylic acid (SA) stress treatments. Moreover, the potential function of *GmVOZ1G* in the response of soybean to drought and salt stress was further investigated.

## 2. Results

### 2.1. Evolutionary and Phylogenetic Relationship of VOZ Proteins

**Figure 1:**
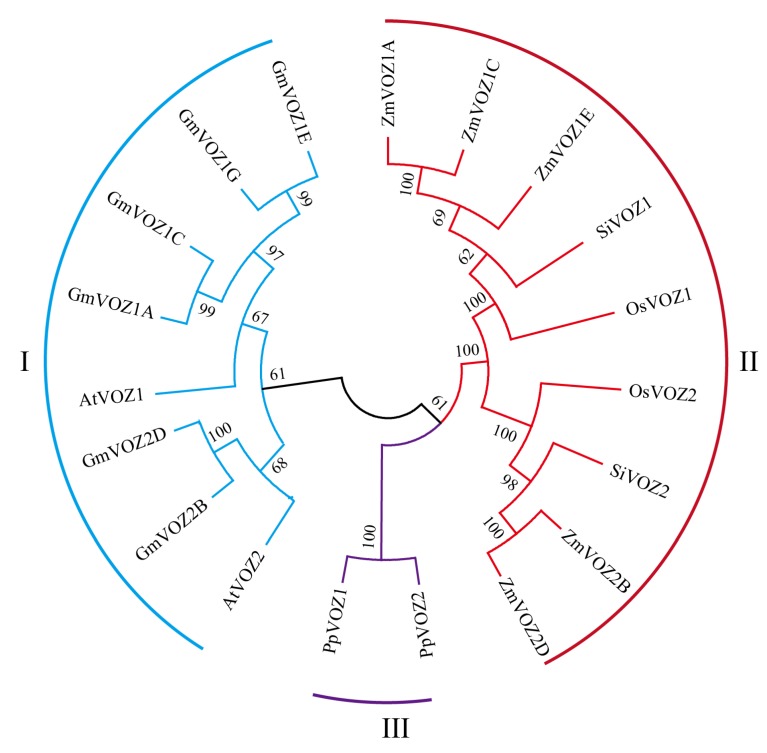
Phylogenetic analyses of vascular plant one-zinc-finger (VOZ) proteins from soybean, *Arabidopsis*, rice, maize, foxtail millet, and *P. patens.* Each cluster (I, II, III) is highlighted in a different color.

Six soybean *VOZ* genes were annotated and designated as *GmVOZ1A, GmVOZ1C, GmVOZ1E, GmVOZ1G, GmVOZ2B,* and *GmVOZ2D* [25]. To elucidate the evolutionary history of soybean *VOZ* genes and their phylogenetic relationship with VOZ family genes in other organisms, VOZ proteins from five other plant species, *Arabidopsis*, rice, maize, foxtail millet, and *P. patens*, were aligned, and then a phylogenetic tree was constructed (Figure 1). All VOZ proteins were divided into three distinct clusters, denoted as cluster I to III. Cluster I contained soybean and *Arabidopsis* VOZ proteins. Cluster II had VOZ proteins from rice, maize, and foxtail millet, and *P. patens* VOZ proteins were assigned to cluster III. Multiple alignments showed that VOZ proteins from soybean, *Arabidopsis*, rice, and *P. patens* shared two conserved domains, termed domain-A and domain-B. Domain-B, containing a zinc finger motif composed of three conserved cysteines and one histidine residue, was also designated as the VOZ-domain (Figure S1) [15].

### 2.2. Gene Structure and Cis-Acting Elements

**Figure 2:**
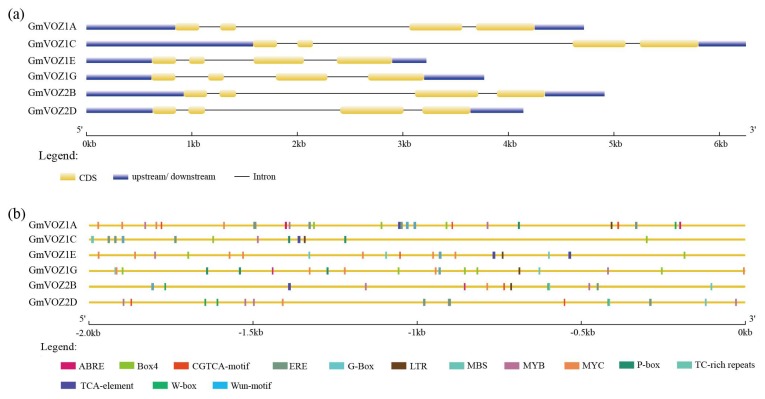
Analysis of exon/intron structures and *cis*–acting elements. (**a**). Exon/intron structures of soybean *VOZ* genes were created with GSDS. Lengths of introns and exons are shown proportionally. (**b**). Putative *cis*-acting elements in a 2.0 kb 5’ flanking region upstream from the start codon of *VOZ* genes in soybean.

As an evolutionary relic, the exon–intron organization might provide useful information for the evolution of a gene family. As shown in Figure 2a, all *GmVOZ* genes, interrupted by three introns, displayed similar exon–intron boundaries. To determine *cis*–acting elements in the promoter region of *GmVOZ* genes, an analysis of *cis*–acting elements was performed in the database PlantCARE. The results showed that *GmVOZ* genes carried abundant stress-related cis–acting elements in their promoters, such as ABRE, G–Box, LTR, MYB, MYC, MBS, TCA–element, CGTCA–motif, W-box or TC-rich repeats, and the numbers varied from one to five. Additionally, two or more light-related *cis*–regulatory Box4 elements were searched in the promoters of *GmVOZ1A, GmVOZ1C, GmVOZ1E,* and *GmVOZ1G* (Figure 2b). The analysis of *cis*–elements in the promoter of *GmVOZ* genes contributed to predicting their biological functions.

### 2.3. Subcellular Localization of GmVOZ Proteins

**Figure 3:**
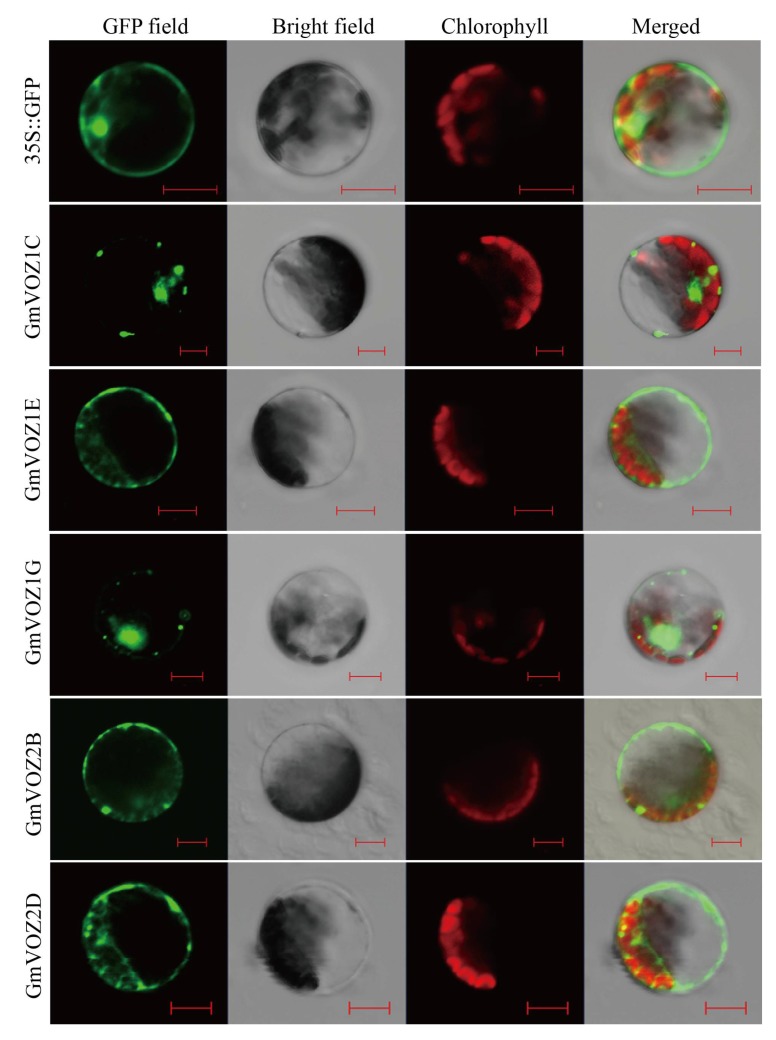
Subcellular localization of soybean VOZ proteins. The recombinant plasmids of GmVOZs–GFP were transformed into Arabidopsis protoplasts. Results were visualized with confocal microscopy 18 h after transformation. Scale bars = 10 μm.

The subcellular localization of a protein is closely related to its biological function [27]. To investigate the subcellular distribution of GmVOZ proteins, the open reading frames (ORFs) of *GmVOZ* genes, lacking the stop codon, were fused to the N-terminal end of the GFP reporter gene. The recombinant GmVOZs–GFP fused plasmids were transformed into *Arabidopsis* protoplasts and monitored by confocal microscopy. It was observed that GmVOZ1E, GmVOZ2B, and GmVOZ2D were located primarily in the cytosol, and the GFP fluorescence of GmVOZ1G fusion protein mainly appeared in the nucleus. GmVOZ1C was probably located in the nucleus, and the possibility of localization in other organelles, such as endoplasmic reticulum (RE), mitochondria or chloroplasts, could also not be ruled out. This is uncertain due to the lack of the corresponding control (Figure 3). Additionally, the fluorescence of GmVOZ1A fusion protein was not detected in *Arabidopsis* protoplasts cells (Figure 3). 

### 2.4. Transcriptional Activation Activity Analysis of GmVOZ Proteins

**Figure 4:**
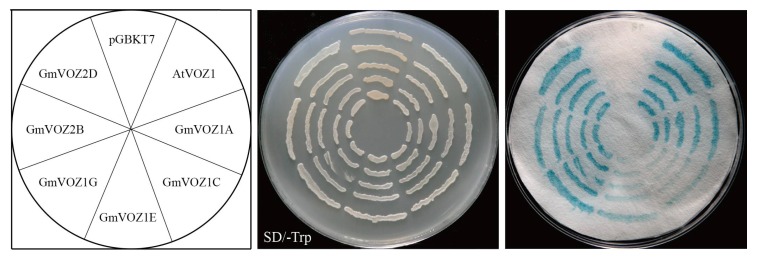
Transcriptional activation activity of soybean VOZ proteins in yeast cells. pGBKT7 and pGBKT7-AtVOZ1 were used as negative and positive controls, respectively.

The transcriptional activation activity of GmVOZ proteins was analyzed using a yeast transactivation system. *GmVOZ* genes were subcloned into a yeast GAL4-DNA binding domain expression vector (pGBKT7), which was subsequently transformed into yeast strain AH109, and then the ability of GmVOZ proteins to activate the transcription of the LacZ reporter gene was detected. As shown in Figure 4, the yeast strain cells harboring the different recombinant plasmids grew well on selective medium SD/-Trp. When we used X–α–Gal, it was discovered that the positive control pGBKT7-AtVOZ1 and the cells containing pGBKT7-GmVOZs showed β–galactosidase activity, whereas the negative control pGBKT7 had no β–galactosidase activity, indicating that soybean VOZ proteins possessed transcriptional activation activity in yeast cells. This finding is in accordance with a previous report on the transactivation activity of VOZ proteins in *Arabidopsis* [15]. Moreover, GmVOZ1C and GmVOZ1E displayed relatively lower levels of transcriptional activation activity, as determined by their ability to activate the expression of the LacZ reporter gene (Figure 4).

### 2.5. Expression Pattern of VOZ Genes in Different Soybean Tissues

**Figure 5:**
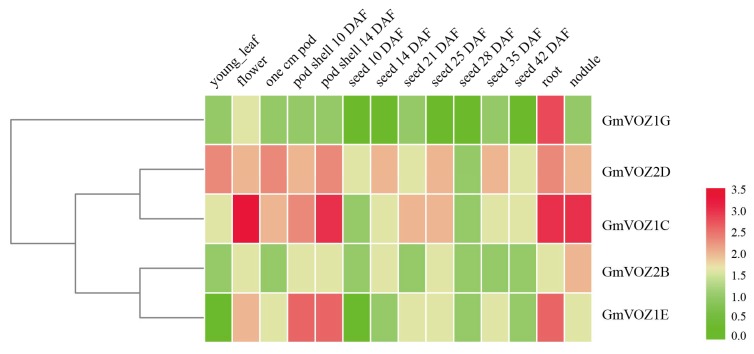
Expression analysis of soybean *VOZ* genes at different developmental stages in specific organs. Data was extracted from the SoyBase database [28].

A RNA–Seq atlas of *Glycine max* was used to identify the expression pattern of *GmVOZ* genes in different tissues [28]. Fourteen tissues at different stages were selected for analysis, including a young leaf, flower, root, nodule, and various stages of pod and seed development. *GmVOZ* genes were expressed in nearly all tissues tested, while the basal expression of *GmVOZ1A* was not detected in the RNA–Seq atlas (Figure 5). The results revealed that distinct *GmVOZ* genes exhibited high variation in their expression profiles, dependent on tissue/organ types and developmental stages. *GmVOZ2D* had higher expression levels in the young leaf, pod, and root, while *GmVOZ1E* had no expression in the young leaf or seed at 10 DAF (days after flowering) but was highly expressed in the root. *GmVOZ1C* was expressed most strongly in the flower. *GmVOZ2B* transcription was most enriched in the nodule. *GmVOZ1G* showed higher expression level in the root but no expression in 10-, 14-, 25-, 28-, and 42-DAF seed (Figure 5). The above results show that VOZ transcription factors may play diverse roles in the growth and developmental regulation of soybean.

### 2.6. Expression Profiles of GmVOZ Genes in Responses to Abiotic and SA Stress

**Figure 6:**
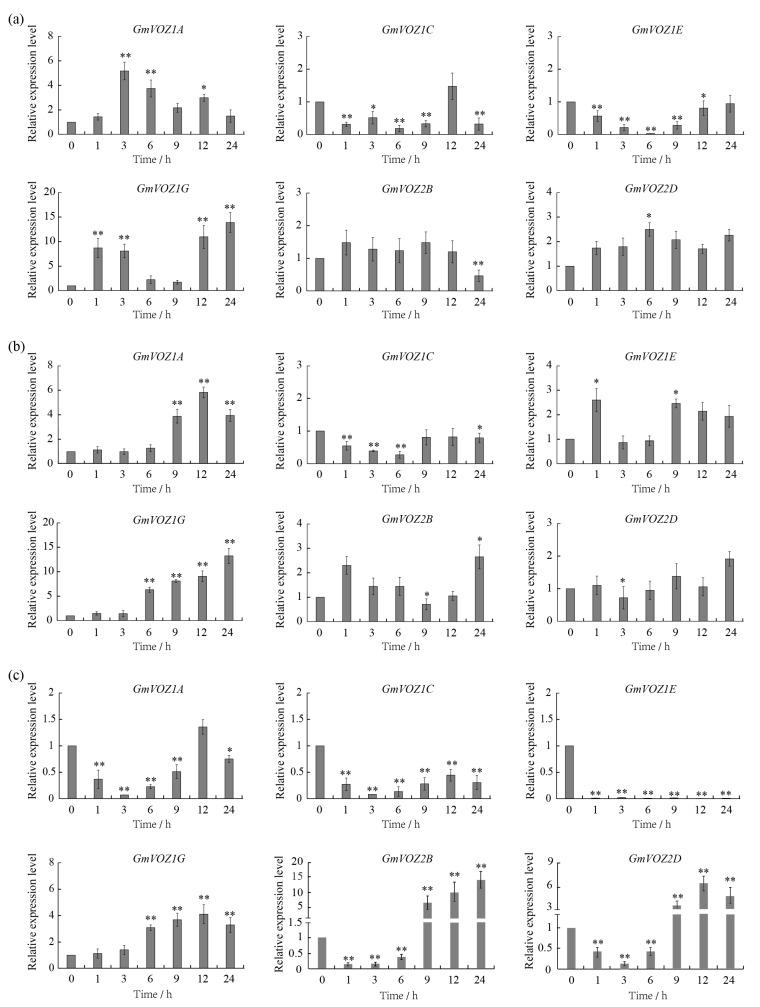
Expression profiles of soybean *VOZ* genes under dehydration, salt, and salicylic acid (SA) treatments. Quantitative real-time PCR (qRT-PCR) analysis of *VOZ* genes in soybean seedlings in response to dehydration (a), NaCl (b), and SA (c). The relative transcription levels of *GmVOZ* genes were normalized to the expression of tubulin. The data are presented as means ± SD of four biological replicates. The asterisks indicate statistical differences in comparison with the corresponding controls at p < 0.01 (**) and 0.01 < P < 0.05 (*), respectively.

To explore the possible involvement of individual *GmVOZ* genes in response to abiotic and biotic stress, the expression patterns of *GmVOZ* genes were initially detected by quantitative real-time PCR (qRT–PCR) in leaves under dehydration, high salinity, and SA stress treatments. Expression data showed that four *GmVOZ* genes had obviously different transcriptional responses to dehydration. Among these genes, the transcription levels of *GmVOZ1A* and *GmVOZ1G* were significantly upregulated and reached a maximum at 3 h (5.2-fold) and 24 h (13.9-fold), respectively. Conversely, *GmVOZ1C* and *GmVOZ1E* were significantly downregulated post treatment. *GmVOZ2B* and *GmVOZ2D* showed a slight response to dehydration stress (increased <3.0-fold). All *GmVOZ* genes were involved in high salinity stress responses (Figure 6a). Similar to dehydration stress treatment, the expression levels of *GmVOZ1A* and *GmVOZ1G* were significantly upregulated after high salinity treatment, and the highest transcription levels occurred at 12 h and 24 h, which were equivalent to 5.8-fold and 13.3-fold increases, respectively. The expression of *GmVOZ1C* showed a significant decrease post high-salinity treatment. Although they participated in the salt stress response, the expressions of *GmVOZ1E, GmVOZ2B*, and *GmVOZ2D* showed weak changes (increased <3.0-fold or 2.0-fold) (Figure 6b). Following SA stress treatment, *GmVOZ1A, GmVOZ1C*, and *GmVOZ1E* were significantly downregulated, especially *GmVOZ1E*; *GmVOZ2B* and *GmVOZ2D* were initially down-regulated at 1-6 h, then began to be significantly up-regulated at 9 h after treatment was initiated, and reached a peak at 24 h (14.2-fold) and 12 h (7.2-fold), respectively. In terms of *GmVOZ1G,* the expression level was increased >4.0-fold after treatment (Figure 6c). These observations suggest that soybean VOZ transcription factors may play diverse roles in plant responses to abiotic and biotic stresses.

### 2.7. GmVOZ1G Positively Regulates Drought and Salt Stress Tolerance in Soybean Hairy Roots

**Figure 7:**
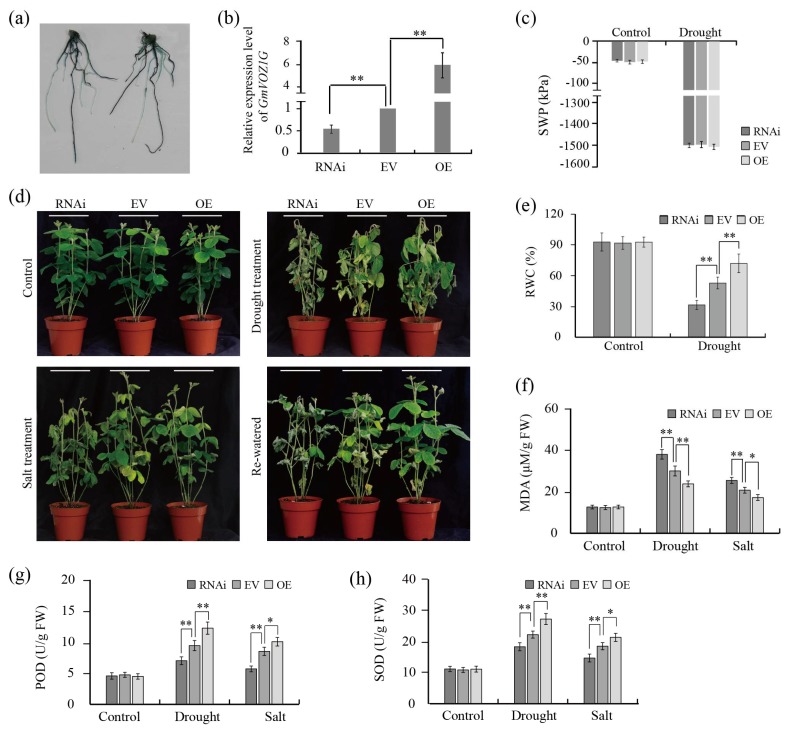
Assessment of the effect of drought and salt stress on *GmVOZ1G* transgenic soybean hairy roots. (**a**). GUS detection of the transformation efficiency in soybean hairy roots. (**b**). qTR–PCR analysis of *GmVOZ1G* transcription levels in overexpression of *GmVOZ1G*, RNAi, and empty vector (EV) control soybean hairy roots. The expression level of tubulin was used as a quantitative control. The means of four biological replicates and the standard deviation are presented. (**c**). Soil water potential (SWP) was determined under normal and drought stress conditions. (**d**). Phenotypes of soybean composite seedlings with overexpression, RNAi, and EV control hairy roots under drought and salt stress treatments. (**e**−**h**). Relative water content (RWC), malondialdehyde (MDA) content, peroxidase (POD), and superoxide dismutase (SOD) activities in leaves of soybean composite seedlings with different transgenic hairy roots under normal and stress conditions. All values are the means and SD of three independent replicates. The asterisks indicate statistical differences in comparison with the corresponding controls at *p* < 0.01 (**) and 0.01 < *p*< 0.05 (*), respectively.

In view of the significance of the response to dehydration and high salinity stress treatments, we hypothesized that *GmVOZ1G* might play an important role in regulation during drought and salt stress responses. Thus, the role of *GmVOZ1G* in drought and salt-stress tolerance was examined by conducting corresponding stress assays in Agrobacterium rhizogenes–mediated transformation of soybean hairy roots [29]. The transformation efficiency of hairy roots was estimated by histochemical GUS staining. As shown in Figure 7a, the ratio of positive hairy roots was relatively high (>60%) in each soybean composite plant. qRT–PCR analysis showed a higher accumulation of *GmVOZ1G* transcription in hairy roots overexpressing *GmVOZ1G* and a lower expression level in RNAi hairy roots compared with the empty vector (EV) control (Figure 7b). After using the above transformation method, a soybean composite plant consisting of a wild-type shoot with transgenic hairy roots is generated [29]. The expression of *GmVOZ1G* was also detected by qRT–PCR in leaves and shoot of soybean composite plants. The results showed that the expression of *GmVOZ1G* had no obvious changes in leaves/shoot of soybean composite plants with overexpression or RNAi hairy roots compared with the EV control (data not shown).

In order to test whether *GmVOZ1G* overexpression or inhibition could change the response of hairy roots to abiotic stress, the drought and salt tolerances of soybean composite plants with transgenic hairy roots were examined. After 12 days of withholding watering, the obvious phenotypic differences were observed among different composite soybean lines. Meanwhile, soil water potential (SWP) was determined. As shown in Figure 7c, SWP had no significant difference among different composite soybean lines under control and stress conditions, and SWP was reduced to approximately –1500 kPa under water deficit condition, this indicates that plants were subjected to severe drought stress [30]. In the absence of stress, there were no apparent differences in shoots among composite seedlings with RNAi, EV control, and the overexpression of hairy roots. Although leaves from composite seedlings were curled, bleached, and withered after 12 days of drought treatment, composite seedlings carrying the RNAi hairy roots were the most severely damaged, followed by the EV-control hairy roots; the least severely damaged were the overexpressed hairy roots. After being re-watered and grown under normal conditions for three days, the majority of leaves from composite seedlings with an overexpression of hairy roots returned to being green, whereas few composite seedlings with RNAi hairy roots were able to do this. Compared with the EV control lines, composite seedlings carrying RNAi hairy roots were more sensitive to salt stress, while overexpressed hairy roots were not. The leaves from composite seedlings with RNAi hairy roots wilted under salt stress for 12 days, and leaves gradually yellowed in composite seedlings with EV-control hairy roots, whereas leaves remained green in composite seedlings with overexpressed hairy roots (Figure 7d). Relative water content (RWC) in plants determines sensitivity to water stress [31]. It was observed that RWC in leaves of soybean composite plants with RNAi hairy roots was significantly lower, and markedly higher in overexpression lines than in the EV-control lines after eight days of drought treatment (Figure 7e).

Malondialdehyde (MDA), superoxide dismutase (SOD), and peroxidase (POD) are important indicators reflecting cell oxidative damage [32]. To explore the potential physiological mechanisms of *GmVOZ1G* overexpression or RNAi hairy roots in enhancing or decreasing drought and salt tolerance, changes in the MDA content and SOD and POD activities in leaves of soybean composite seedlings carrying RNAi, the EV control, and overexpressed hairy roots with or without stress treatments were investigated. No significant differences in the MDA content and SOD and POD activities were observed in RNAi, the EV control, and overexpression lines under non-stress conditions. Under drought and salt stress for eight days, the MDA content and SOD and POD activities increased in all RNAi, EV-control, and overexpression lines. Compared with the EV control, the MDA content displayed a significantly higher accumulation in RNAi lines, but an obviously lower accumulation was observed in overexpression lines. However, the activities of SOD and POD were dramatically lower in RNAi lines, and markedly higher in overexpression lines (Figure 7f–h). Taken together, these findings demonstrate that *GmVOZ1G* is a positive regulator of plant tolerance to drought and salt stress.

## 3. Discussion

Although *VOZ* genes have been previously classified as members of the VIII-2 subfamily of the NAC transcription factor family, the sequence alignments showed a low homology at the NAC domain between VOZ and NAC proteins in *Arabidopsis* [33]. Moreover, NAC and VOZ proteins are divided into two separate transcription factor families in the PlantRegMap database [26]. Recently, an evolutionary analysis also agreed with the classification of *VOZ* genes as an independent gene family [25]. Compared to soybean, the *VOZ* gene family is relatively small, with two members in the *Arabidopsis*, foxtail millet, rice, *Brachypodium distachyon* or tobacco genome [15,25]. These data indicate that the number of VOZ family genes in soybean has expanded compared with other species as mentioned above; this might be due to two genome duplication events in soybean [34]. Soybean VOZ proteins could be clustered together with VOZ proteins from dicotyledonous *Arabidopsis*, but not with VOZ proteins from monocotyledonous rice, maize, and foxtail millet in the same clade, this demonstrates that VOZ transcription factors have diverged before monocot and dicot plants differentiated. Intron gain or loss is one of the evolutionary mechanisms underlying the genesis of a gene family [35,36,37]. The exon/intron structure analysis revealed that dicotyledonous soybean and *Arabidopsis VOZ* genes shared an analogous intron distribution pattern that was different from that in monocotyledonous rice and foxtail millet (Figure 2a and Figure S2), implying that the diverse status of exon and intron splicing might play a significant role in the evolutionary process of *VOZ* genes between monocots and dicots. 

Although VOZ transcription factors were assumed to function as transcriptional activators [15], VOZ1/2 proteins were dispersed primarily in the cytosol under normal growth conditions [19,22]. Recently, it was reported that VOZ proteins relocated to the nucleus, and subsequently were rapidly degraded via the ubiquitin/26S proteasome system under certain stress conditions [22,24]. Moreover, VOZ proteins translocated from the cytoplasm to the nucleus and participated in the regulation of flowering signaling in *Arabidopsis* [19], suggesting that the nuclear localization of VOZ transcription factors is required for their functions. Interestingly, GmVOZ1G was predominantly located in the nucleus under non-stress conditions (Figure 3), indicating that *GmVOZ1G* might play different regulatory roles in plant growth, development or stress responses compared with VOZ transcription factors located in the cytosol. To a certain extent, this was confirmed by the fact that *GmVOZ1G* acted as a positive regulator in drought stress tolerance (Figure 7). In *Arabidopsis*, VOZ1/2 negatively regulated drought, cold, and high-temperature stress tolerance through the 26S proteasome–mediated degradation of VOZ proteins in the nucleus [21,22]. Experimental evidence is required to confirm whether GmVOZ1G is degraded by the ubiquitin/26S proteasome under non-stress or stress conditions. 

The roles of the phytohormones abscisic acid (ABA), SA, and ethylene (ET) in the adaptation of plants to various abiotic stresses and pathogenic infection have been well characterized [38,39,40,41,42,43,44,45]. VOZ1/2 were also shown to participate in regulating SA- and jasmonic acid (JA)-mediated defense responses to pathogens in *Arabidopsis* [21]. In the current study, our data revealed that individual *GmVOZ* genes possessed at least one ABA-responsive element (ABRE), SA-responsive element (TCA–element) or ET-responsive element (ERE) in their promoter regions (Figure 2b and Table S2). In addition, several stress-related *cis*-acting elements were also identified in the promoter region of *GmVOZ* genes (Figure 2b and Table S2), with evidence demonstrating that these *cis*-elements are involved in responses to drought, salt, low temperature or pathogen attack [46,47,48,49,50,51]. In light of the existence of these *cis*-acting elements, *GmVOZ* genes are proposed to play a broad role in plant responses to abiotic and biotic stresses. This was further supported by the observation that *GmVOZ* genes were involved in the response to dehydration, salt, and SA stress treatments (Figure 6). 

It was proposed that abiotic and biotic stresses mediate the expression of overlapping sets of genes in plants [52]. Our expression data showed that *GmVOZ1G* was significantly upregulated by dehydration, salt, and SA stress treatments, whereas *GmVOZ1C* was downregulated. Other *GmVOZ* genes displayed diverse response mechanisms under different stress treatments (Figure 6). Interestingly, *GmVOZ1* genes showed a significant alteration in the expression levels in response to dehydration and salt stress treatments, and the transcript levels of *GmVOZ2* genes showed a weak change. In response to SA stress treatment, the expression levels of *GmVOZ1* genes were significantly decreased, except for *GmVOZ1G*, whereas *GmVOZ2* genes were obviously upregulated (Figure 6). These observations suggest that GmVOZ2 transcription factors might be involved specifically in the response to biotic stress, and GmVOZ1 and GmVOZ2 transcription factors likely play diverse roles in biotic stress responses. More work is needed to investigate the specific function of soybean VOZ transcription factors in response to abiotic and biotic stresses.

VOZ transcription factors were isolated as proteins that bind to a special *cis*-acting region responsible for the pollen-specific gene expression of *AVP1* [15,53]. In *Arabidopsis*, VOZ1/2 have also been shown to play essential roles in regulating flowering time [16,17,19]. Here, valuable information was provided on the conserved functional roles of *VOZ* genes by phylogenetic data and multiple sequence alignments of VOZ proteins from soybean and other species [Figure 1, Figure S1]. Thus, it is possible that soybean VOZ transcription factors participate in flowering regulation. Light, as a key environmental factor, affects flowering time [54]. In the present study, the *cis*-acting element analyses showed that *GmVOZ1* genes contained two or more light-related *cis*-elements of Box4 in their promoter regions [55]. Additionally, the expression patterns in different soybean tissues showed that *GmVOZ1C* transcription was most enriched in flowers. These observations indicate that GmVOZ1 transcription factors might be required for the regulation of flowering. Nevertheless, no evidence was provided here to determine whether GmVOZ2 transcription factors are involved in flowering time control. More experimental evidence is needed to uncover the role of soybean VOZ transcription factors in regulating flowering. 

## 4. Materials and Methods 

### 4.1. Sequence Alignment and Phylogenetic Analysis

The peptide sequences of the VOZ protein from different species were obtained from the Phytozome (version 12.1) database. The ClustalX (version 2.1) program was used to perform multiple sequence alignments of the VOZ family protein [56]. The phylogeny was obtained by the neighbor-joining method using MEGA6.0 software with the following parameters: 1000 bootstrap replicates [57].

### 4.2. Gene Structure and Potential Cis–Acting Element Analysis

The coding and genomic DNA sequences of the soybean *VOZ* gene were retrieved from the Phytozome database. The exon–intron arrangements of soybean *VOZ* genes were defined using the online program Gene Structure Display Server2.0 (GSDS) by comparing coding sequences and the corresponding genomic DNA sequences [58]. The 2.0 kb promoter sequences upstream of the soybean *VOZ* gene transcription initiation site were extracted from the Phytozome database. Potential *cis*-acting elements of extracted promoter regions were analyzed using the PlantCARE database [59].

### 4.3. Sub-cellular Localization and Transcriptional Activation Analysis

The coding regions of the soybean *VOZ* gene were amplified and subcloned into the 16318GFP expression vector using the *Bam*HI restriction site. The reconstructed VOZs–GFP fused plasmids were transferred into *Arabidopsis* protoplasts using the PEG4000-mediated transformation method, and the empty 16318GFP vector was used as the control. The GFP signal was detected by a laser scanning confocal microscopy (Zeiss LSM 700, Germany) after incubating in darkness at 22 °C for 18 h [60].

The full-length cDNA sequences of the soybean *VOZ* gene and *Arabidopsis VOZ1* were individually amplified and fused into the pGBKT7 vector using the *Eco*RI restriction site to assess protein transcriptional activity in yeast cells. pGBKT7-AtVOZ1 and pGBKT7 were used as the positive and negative control, respectively. The reconstructed plasmids were transformed into yeast host strain AH109 using YeastmakerTM Yeast Transformation System 2 kit (Clontech, USA) according to the manufacture’s protocol. Transcriptional activity was analyzed according to the methods established in prior work [61]. Yeast cells harboring the reconstructed plasmids were cultivated on selective media SD/-Trp [0.67% (*m/v*) yeast nitrogen base, 2% (*m/v*) glucose, and 0.074% (m/v) -Trp DO Supplement (Clontech, USA), pH 5.8−6.0] for three days at 30 °C. Yeast strain grown on selective media SD/-Trp were transferred to filter paper and frozen with liquid nitrogen, and subsequently incubated with a Buffer/X–α–Gal solution [0.59 mM Na_2_HPO_4_·7H_2_O, 0.35 mM NaH_2_PO_4_·H_2_O, 0.098 mM KCl, 0.33% (*v/v*) β–mercaptoethanol, and 0.34 mg mL^−^^1^ X–α–Gal] for 8 h at 30 °C. The primers are listed in Supplementary Table S1.

### 4.4. Expression Patterns of GmVOZ Genes in Different Tissues

The expression analysis of soybean *VOZ* genes in different tissues and development stages was conducted using gene transcript abundance values obtained from the SoyBase database [28]. The heatmap was produced by TBtools software [62].

### 4.5. Plant Materials and Stress Treatments

Soybean variety Williams 82 and *Arabidopsis* plants of the Col-0 ecotype were used throughout this study. Soybean seedlings were grown in pots containing a mixture of humus and vermiculite (1:2, *v/v*) in a greenhouse at 26 °C with a 16-h-light/8-h-dark photoperiod and 60% relative humidity. *Arabidopsis* plants were grown in mixed soil (humus:vermiculite = 1:1) in a growth chamber under a 16-h-light/8-h-dark photoperiod at temperature of 24 °C. Fifteen-day-old soybean seedlings were exposed to different stress treatments. Stress treatments were performed as follows: for dehydration, soybean seedlings were removed from the soil and then placed on filter paper under 60% relative humidity at room temperature (22 °C). For salt treatment, the roots of seedlings were subjected to 200 mM NaCl solution. SA stress treatment was performed as described previously [63]. The leaves under different treatments were sampled for RNA extraction at 0, 1, 3, 6, 9, 12, and 24 h. 

### 4.6. RNA Extraction and Quantitative Real-time PCR (qRT–PCR)

Total RNA was extracted from plant samples using Trizol solution (TaKaRa, Japan), and first-strand cDNA was reverse-transcribed using the PrimeScript 1st Strand cDNA Synthesis Kit (TaKaRa, Japan). qRT–PCR analysis was conducted with an ABI prism 7500 Real-Time PCR system (Applied Biosystem, Life Technologies, Carlsbad, CA, USA) using Top Green qPCR SuperMix (+ Dye I) kit (TransGen, Beijing, China). PCR was performed after a preincubation at 94 °C for 30 s and was followed by 40 cycles of amplification (94 °C for 5 s, 58 °C for 15 s, and 72 °C for 32 s). Each sample with four biological replicates was quantified using the 2^−^^ΔΔCt^ method according to the cycle threshold (*Ct*) values [64]. The transcript level of the soybean *tubulin* gene (Glyma.03g124400) was used as an internal control. All primer sequences were designed using Primer Premier 5.0 software and are detailed in Table S1. 

### 4.7. Agrobacterium Rhizogenes–Mediated Transformation of Soybean Hairy Roots

For the overexpression vector construction, the coding sequence of *GmVOZ1G* was amplified and cloned into the pCAMBIA3301 expression vector using the *Nco*I and *Bst*EII restriction sites, resulting in the construct pCAMBIA3301-GmVOZ1G. In order to generate the RNAi suppression vector pCAMBIA3301-GmVOZ1G-RNAi, a 666 bp ligated sequence, containing a *GmVOZ1G* fragment (from positions -23 to 227) and its antisense fragment, with a fragment of intron 1 of maize alcohol dehydrogenase-1 (Adh1) between the two *GmVOZ1G* fragments, was synthesized (Biomed, Beijing, China) and inserted into the pCAMBIA3301 vector using the *Nco*I and *Bst*EII restriction sites. The primers and sequence used in this assay are listed in Table S1. The constructs were transformed into hairy roots using a high-efficiency *A. rhizogenes*-mediated genetic transformation method established in prior work [29]. Seven-day-old seedlings with unfolded cotyledons were injected with *A. rhizogenes* strain K599 harboring the reconstructed plasmids at the cotyledonary node. Subsequently, the infected seedlings were transferred into vermiculite and kept in a high humidity and temperature condition. When hairy roots generated at the infection site and could support the plants (approximately 5–10 cm in length), the primary roots were removed and the transgenic hairy roots were tested by qRT–PCR. Soybean composite seedlings consisting of transgenic hairy roots were transplanted into nutritious soil (humus:vermiculite = 1:2) and cultured in 16-h-light/8-h-dark photoperiod at 28 °C in a greenhouse. Finally, 119 (OE), 132 (RNAi), and 165 (EV control) soybean composite plants with similar growth patterns and expression level of *GmVOZ1G* in hairy roots were selected for further study, respectively. 

For drought treatment, water was withheld from two-week-old soil-grown soybean composite seedlings for 12 days, after which they were re-watered and allowed to recover for three days. For salt treatment, two-week-old soil-grown soybean composite seedlings were subjected to treatment with 200 mM NaCl solution for 12 days. The content of MDA, SOD, and POD activities in leaves were measured eight days after drought and salt treatments with the corresponding test kits (Cominbio, Suzhou, China) according to the manufacture’s protocols. All experiments were repeated independently at least three biological replicates.

### 4.8. GUS Staining

For GUS histochemical staining assay, the transgenic hairy roots (EV control) were incubated with a GUS staining solution [1 mg mL^−1^ 5-bromo-4-chloro-3-indolyl β–D–glucuronic acid, 1.0 mM K_3_Fe(CN)_6_, 1.0 mM K_4_Fe(CN)_6_, 50 mM Na_2_HPO_4_ buffer, pH 7.0, 10 mM EDTA, and 0.1% (*v/v*) TritonX-100] (Real Times, Beijing, China) for 16 h at 37 °C, followed by treating with 70% ethanol for 8 h.

### 4.9. Measurement of Soil Water Potential (SWP) and Relative Water Content (RWC)

SWP was measured using WP4-T Dewpoint meter (Decagon Devices, Pullman, WA, USA) according to the manufacturer’s instructions [30]. RWC was determined in leaves of soybean composite plants as described previously [30,65].

### 4.10. Statistical Analysis

A Student’s t test analysis was performed in Microsoft Excel 2007. Data are shown as means ± standard deviation (SD), with a P-value cut-off of 0.05.

## 5. Conclusions

An extensive investigation of six soybean *VOZ* genes was conducted in terms of their phylogeny, sequence, expression analyses, and responses to abiotic stress. The observations demonstrate that soybean *VOZ* genes act as important components of stress response and have complex regulatory mechanisms in plants. Our study provides valuable information for a more detailed analysis of the biological function of *VOZ* genes in soybean.

## Supplementary Materials

Supplementary materials can be found at https://www.mdpi.com/1422-0067/21/6/2177/s1. Figure S1. Alignments of VOZ proteins from soybean, Arabidopsis, rice, and P. patens. Black lines indicate two conserved domains. Red boxes indicate conserved residues possibly forming functional zinc-coordinating motifs. Figure S2. Intron/exon structures of VOZ genes in Arabidopsis, rice, and foxtail millet. Table S1. The primers and sequences used in this study. Table S2. Cis–acting elements in the promoters of soybean *VOZ* genes.

## Abbreviations

VOZ	Vascular plant One-Zinc finger
MDA	Malondialdehyde
POD	Peroxidase
SOD	Superoxide dismutase
qRT–PCR	Quantitative real-time PCR
GFP	Green fluorescent protein
SA	Salicylic acid
ORF	Open reading frame
FW	Fresh weight
EV	Empty vector
