# Supplementary figures and images for: Expression Analyses of Soybean VOZ Transcription Factors and the Role of GmVOZ1G in Drought and Salt Stress Tolerance
Source: Int J Mol Sci. 2020 Mar 21;21(6):2177. doi: 10.3390/ijms21062177 (PMC7139294; doi:10.3390/ijms21062177)

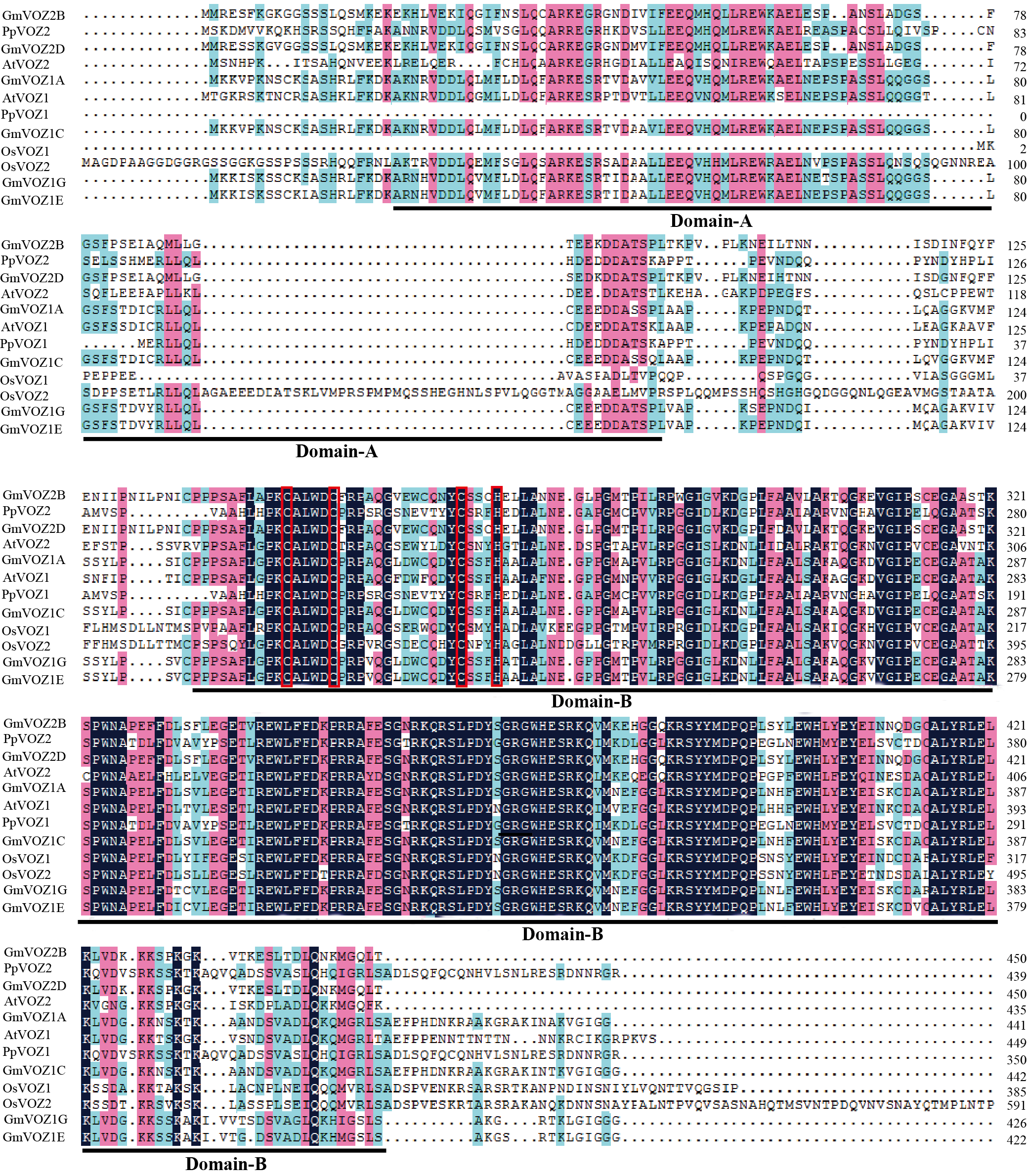

Supplement: Supplementary file 1 [file ijms-21-02177-s001.zip › supplementary materials/Figure S1.tif]

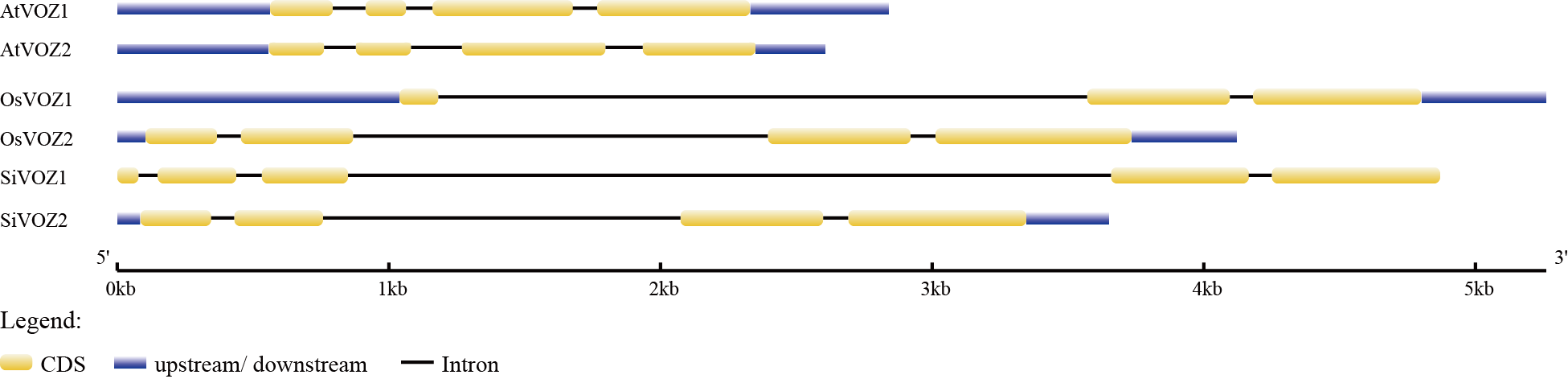

Supplement: Supplementary file 1 [file ijms-21-02177-s001.zip › supplementary materials/Figure S2.tif]
